# A cross-country study on the impact of governmental responses to the COVID-19 pandemic on perinatal mental health

**DOI:** 10.1038/s41598-023-29300-w

**Published:** 2023-02-16

**Authors:** Ana Mesquita, Raquel Costa, Rena Bina, Carmen Cadarso-Suárez, Francisco Gude, Carla Díaz-Louzao, Pelin Dikmen-Yildiz, Ana Osorio, Vera Mateus, Sara Domínguez-Salas, Eleni Vousoura, Drorit Levy, Samira Alfayumi-Zeadna, Claire A. Wilson, Yolanda Contreras-García, Mercedes Carrasco-Portiño, Sandra Saldivia, Andri Christoforou, Eleni Hadjigeorgiou, Ethel Felice, Rachel Buhagiar, Camellia Hancheva, Erilda Ajaz, Ana Uka, Emma Motrico

**Affiliations:** 1 School of Psychology, University of Minho, Campus Gualtar, 4710-057 Braga, Portugal; 2 EPIUnit - Instituto de Saúde Pública, Universidade do Porto, Rua das Taipas,n° 135, 4050-600 Porto, Portugal; 3 Laboratório para a Investigação Integrativa e Translacional em Saúde Populacional (ITR), Universidade do Porto, Rua das Taipas, n° 135, 4050-600 Porto, Portugal; 4 Hei-Lab: Digital Human-Environment Interaction Lab, Faculty of Psychology, Education and Sports, Lusófona University, Porto, Portugal; 5 School of Social Work, Bar Ilan University, Ramat Gan, Israel; 6 Department of Statistics, Mathematical Analysis, and Optimization, Group of Biostatistics and Biomedical Data Science, University of Santiago de Compostela, Santiago de Compostela, Spain; 7 Department of Epidemiology, Research Group On Epidemiology of Common Diseases, University Clinical Hospital of Santiago, Santiago de Compostela Health Research Institute (IDIS), Santiago de Compostela, Spain; 8 Department of Psychology, Kirklareli University, Kirklareli, Turkey; 9 Graduate Program on Developmental Disorders and Mackenzie Center for Research in Childhood and Adolescence, Center for Biological and Health Sciences, Mackenzie Presbyterian University, São Paulo, Brazil; 10 Psychology Department, Universidad Loyola Andalucia, Sevilla, Spain; 11 Department of Psychology, School of Philosophy, National and Kapodestrian University of Athens, Athens, Greece; 12 The Center for Women’s Health Studies and Promotion, Ben-Gurion University of the Negev, Beersheba, Israel; 13 Nursing Department, School of Health Sciences, Ashkelon Academic College, 78682 Ashkelon, Israel; 14 Section of Women’s Mental Health, King’s College London and South London and Maudsley NHS Foundation Trust, London, UK; 15 Department of Obstetrics and Puericulture. Faculty of Medicine, Universidad de Concepción, Concepción, Chile; 16 Department of Psychiatry and Mental Health. Faculty of Medicine, Universidad de Concepción, Concepción, Chile; 17 Department of Social and Behavioral Sciences, European University Cyprus, Engomi, Cyprus; 18 Department of Nursing, School of Health Science, Cyprus University of Technology, Limassol, Cyprus; 19 Department of Psychiatry, University of Malta, Msida, Malta; 20 Sofia University “St. Kliment Ochridski”, Sofia, Bulgaria; 21 Department of Education and English Language, Beder University College, Tirana, Albania; 22 Present Address: ProChild CoLAB, Campus de Azurém, Guimarães, Portugal

**Keywords:** Psychology, Human behaviour

## Abstract

This study aimed to analyse the role of governmental responses to the coronavirus disease 2019 (COVID-19) outbreak, measured by the Containment and Health Index (CHI), on symptoms of anxiety and depression during pregnancy and postpartum, while considering the countries’ Inequality-adjusted Human Development Index (IHDI) and individual factors such as age, gravidity, and exposure to COVID-19. A cross-sectional study using baseline data from the Riseup-PPD-COVID-19 observational prospective international study (ClinicalTrials.gov: NCT04595123) was carried out between June and October 2020 in 12 countries (Albania, Brazil, Bulgaria, Chile, Cyprus, Greece, Israel, Malta, Portugal, Spain, Turkey, and the United Kingdom). Participants were 7645 pregnant women or mothers in the postpartum period—with an infant aged up to 6 months—who completed the Edinburgh Postnatal Depression Scale (EPDS) or the Generalised Anxiety Disorder Assessment (GAD-7) during pregnancy or the postpartum period. The overall prevalence of clinically significant depression symptoms (EPDS ≥ 13) was 30%, ranging from 20,5% in Cyprus to 44,3% in Brazil. The prevalence of clinically significant anxiety symptoms (GAD-7 ≥ 10) was 23,6% (ranging from 14,2% in Israel and Turkey to 39,5% in Brazil). Higher symptoms of anxiety or depression were observed in multigravida exposed to COVID-19 or living in countries with a higher number of deaths due to COVID-19. Furthermore, multigravida from countries with lower IHDI or CHI had higher symptoms of anxiety and depression. Perinatal mental health is context-dependent, with women from more disadvantaged countries at higher risk for poor mental health. Implementing more restrictive measures seems to be a protective factor for mental health, at least in the initial phase of the COVID-19.

## Introduction

After the World Health Organization’s declaration of the coronavirus disease 2019 (COVID-19) global pandemic on March 11th, 2020, a wide range of non-pharmaceutical interventions were rapidly implemented by governments in many countries^1,2^, in an attempt to reduce the burden of the disease and allow healthcare systems to better prepare and respond^3^. These measures, which included restrictions on social contact and activities, had significant economic and societal impacts, namely in terms of mental health^4,5^, and health-related quality-of-life^6^ and therefore should be considered to fully understand the impact of the COVID-19 pandemic on global mental health.

Exposure to natural disasters or stressful life events are major contributors to mental health disorders among pregnant and postpartum women^7^, highlighting the importance of mental health surveillance during the current COVID-19 pandemic, as exacerbation of negative emotional outcomes in these women may be expected^8,9^. Studies carried out during the pandemic have reported higher rates of prenatal depression varying from 25 to 45,4%^10–14^, and as high as 43% for the postpartum period^11,15^ compared with the pre-pandemic period. Additionally, the prevalence of anxiety almost doubled compared to pre-pandemic rates^16–18^, ranging from 30,5 to 48,8% in pregnant women^10–14^, and as high as 61% in the postpartum period^12,13,15^.

Monitoring anxiety and depression in the perinatal period is extremely important because it is linked not only to adverse impacts on mothers’ well-being and physical health^19^ but also to suboptimal child development, including compromised physical and cognitive development, behavioural problems, and increased risk of mental disorders^20^.

Importantly, not all women seem to be equally affected^21^, with a higher risk for perinatal mental disorders found among those living in socioeconomically disadvantaged contexts^22,23^, highlighting the influence of socioeconomic inequalities on perinatal mental health during the pandemic.

Therefore, this study aimed to analyse for the first time the role of governmental measures—using the Containment and Health Index (CHI)—on symptoms of depression and anxiety in pregnant and postpartum women, while considering countries’ Inequality-adjusted Human Development Index (IHDI) scores and individual factors such as age, gravidity, and COVID-19 exposure.

## Methods

### Study design and setting

#### Setting

This was a cross-sectional study using baseline data from the Riseup-PPD-COVID-19 observational prospective international study (ClinicalTrials.gov: NCT04595123). The method has been previously described in detail elsewhere^24^. Briefly, pregnant and postpartum women were invited to participate in the study through social media, networks of organisations (including universities, health care services, perinatal mental health non-government organisations), policymakers, local organisations, and other stakeholders, as well as networks of colleagues and acquaintances of the research teams. Participants were also recruited directly by message or email (only researcher’s personal contacts were targeted).This study received ethical approvals in each country from which data was gathered and has been performed in accordance with the latter amendments of Declaration of Helsinki and with the Regulation (EU) 2019/679 of the European Parliament and the Council of April 27 2016 on the protection and transfer of personal data (GDPR).

The study was carried out in 12 countries, Albania (n = 37), Brazil (n = 865), Bulgaria (n = 84), Chile (n = 444), Cyprus (n = 469), Greece (n = 713), Israel (n = 549), Malta (n = 263), Portugal (n = 1422), Spain (n = 828), Turkey (n = 1426), and the United Kingdom (n = 545), from June to October 2020.

#### Participants

Pregnant women or mothers in the postpartum period—with an infant aged up to 6 months—were eligible to participate in the study. Further inclusion criteria were: (1) at least 18 years of age and (2) living in one of the 12 eligible countries.

Between June 7th and October 31st, 2020, 15,611 potential participants accessed the questionnaire link. Of these, 1,798 (11.5%) did not provide information regarding the eligibility criteria, and 260 (1.7%) were not eligible for participation after responding to the eligibility questions.

Therefore 13,553 potentially eligible participants gave their informed consent. Of these, 2,965 (21.9%) were excluded due to incomplete questionnaires (e.g., not providing answers beyond the eligibility questions; n = 2553), incongruent data (e.g., date of birth of child indicating that the child is older than 6 months; n = 300) or duplicate responses (verified by checking answers with the same contact information—e.g., email address—and matching sociodemographic data; n = 112). From the remaining 10,588 participants, only women who completed the Edinburgh Postnatal Depression Scale (EPDS) or the Generalised Anxiety Disorder Assessment (GAD-7) were included in this analysis, yielding a final sample of n = 7,645.

#### Measures

The questionnaire was initially developed in English and translated into the participating countries’ languages using back translation, as described in detail elsewhere^24^.

### Sociodemographic characteristics

Sociodemographic information included age, country of birth, country of residence, educational level (secondary education or lower vs. higher education), cohabitation with a partner (no vs. yes), professional status (employed vs. unemployed), gravidity (primigravida vs multigravida), exposure to COVID-19 (no vs. yes if exposed to at least one of the following: history of COVID-19 diagnosis, COVID-19 related symptoms, direct physical contact with an infected person, or death of a relative with COVID-19), and history of mental health problems (no vs. yes if at least one of the following: previous treatment for mental health problems, previous treatment for substance abuse, previous untreated mental health problems, and previous untreated substance abuse).

### Governmental responses to the COVID-19 outbreak and number of deaths due to COVID-19

The CHI developed by Hale, Petherick, Phillips, Webster was used to evaluate the level of governmental response across the COVID-19 pandemic period under assessment^1^ It is calculated based on the following composite of closure, containment, and health measures: schools closing, workplaces closing, cancelled public events, restrictions on gatherings, closing of public transport, stay-at-home requirements, restrictions on in-country movements, international travel restrictions, public information campaigns, testing policies, contact tracing, face coverings, vaccination policies, and protection of older adults. Higher CHI indicates greater levels of government restrictions. In order to assess the women’s exposure to countries’ CHI, we used the area under the curve of the CHI 30 days before the completion of the questionnaires for each woman. The number of deaths per million at the time of questionnaire completion was used to indicate the impact of COVID-19 on each country’s population. We used the information available online at ‘“our world data”^25^.

### Country development index

The United Nations developed the IHDI to capture the distribution among a country’s population of its achievements in health, education, and income, accounting for inequality level. We used the 2019 estimates of the IHDI available online^26^. Higher IHDI indicates better national achievements in these areas.

### Symptoms of depression

The EPDS^27^ was used to assess the depression symptoms. It is a ten-item self-report inventory with a four-point Likert scale. Total scores range from 0 to 30, with higher scores indicating higher symptoms. The threshold of 13 or more was used to indicate clinically significant symptoms of depression^28^, and the reliability was very good (Cronbach’s alpha = 0.881).

### Symptoms of anxiety

GAD-7^29^ was used to assess the severity of anxiety symptoms. This is a seven-item self-report questionnaire with a four-point Likert scale. It was developed based on the Diagnostic and Statistical Manual of Mental Disorders (DSM)-IV and DSM-IV-Text Revision criteria. Total scores range from 0 to 21, with higher scores indicating higher symptoms. The threshold of 10 or more was used to indicate clinically significant anxiety symptoms^16,30^, and the reliability was very good (Cronbach’s alpha = 0.903).

### Statistical analysis

A descriptive analysis was conducted of the country-related and individual-related variables, alongside GAD-7 and EPDS total scores -, as both binary (GAD-7 ≥ 10 vs. GAD-7 < 10; EPDS ≥ 13 vs. EPDS < 13) and continuous variables. Median (Mdn) and interquartile (IQ) range were computed for continuous variables, and absolute frequencies and percentages were computed for categorical variables.

Multivariate analyses were performed separately for pregnant and postpartum women, with GAD-7 or EPDS continuous scores as dependent variables, according to gravidity (primigravida *vs* multigravida), COVID-19 exposure, living with a partner (no *vs* yes), history of mental health problems (no *vs* yes), age (in years), and country-related covariates (CHI, deaths per million, and IHDI), and intra-country correlation (since it is expected that women from the same country are more closely related to each other than to women from other countries). Missing data were not imputed; therefore, 353 women without data on age and three without data on gravidity were not included in the models. The models were fitted using a negative binomial regression model based on Generalized Additive Mixed Models^31^, to consider possible non-linear effects of the continuous variables using thin plates splines. A sensitivity analysis was conducted, not including Albania, Bulgaria, and Malta since these countries had sample sizes < 300^24^.

Descriptive analyses were carried out in statistical software SPSS, version 26.0, while multivariate regression analyses were carried out in the statistical software R (R Core Team, 2020), version 4.1.0, using the GJRM, mgcv, and ggplot2 packages.

### Role of the funding source

The study's funding sources had no role in study design, data collection, data analyses, data interpretation, or report writing.

### Ethical approval

This protocol and the template of informed consent forms were reviewed and approved by the followings Ethics Committees: Bedër University College, Albania (Ethics protocol: 145); Sofia University “St. Kliment Ohridski”, Bulgaria (Ethics protocol approved 21th June 2020); Cyprus National Bioethics Committee (Ethics protocol: EEBK ΕΠ 2020.01.126); France (EThics approval: Ref. N°: 20.11.16.46440/CPP2020–11-100b/2020- A02289–30); American College of Greece (Ethics protocol: #202005207); Bar Ilan University School of Social Work, Israel (Ethics approval 062001); University of Malta (Ethics protocol: FRECMDS_1920_179); University of Minho, Portugal (Ethics Protocol: CEICVS 045/2020); Andalusian Ministry of Health, Spain (Ethics Protocol: 1257- N-20); Kırklareli University, Turkey (Ethics protocol: 35523585–199-E.8606); King’s College London, the United Kingdom (Ethics protocol: ID 19747); National University of Entre Ríos, Argentina (Ethics Protocol: CD 610/09); Mackenzie Presbyterian University, Brazil (Ethics Protocol: 31155120.7.0000.0084); Universidad de Concepcion, Chile (Ethics Protocol: CEC 13/2020 and CEBB 704–2020). Participants gave their informed consent via online, and protection of personal data was ensured by adherence to guidelines and regulations in each country.

## Results

A total of 7,645 women, either pregnant (n = 3,503) or in the postpartum period (n = 4,142) were considered in these analyses. Table 1 shows the participant’s characteristics. Multigravida, either pregnant or in the postpartum period, were older (*p* < 001/*p* < 001), were less exposed to COVID-19 (*p* = .003/*p* < .001), and lower education levels compared with primigravida (*p* = .001/*p* = 019). Postpartum women were older (*p* < 001), were more exposure to COVID-19 (*p* = .031), and lower rates of unemployment (*p* < 001) compared to pregnant women.

**Table 1:** Participants’ characteristics.

		Pregnant women				Postpartum women					
		Primigravida	Multigravida	Total	*p*-value	Primigravida	Multigravida	Total	*p*-value	Total	*p*-value*
		N = 2036^1^ N (%)	N = 1466^1^ N (%)	N = 3503 N (%)		N = 2366^2^ N (%)	N = 1774^2^ N (%)	N = 4142 N (%)		N = 7645 N (%)	
Women age					< .001				< .001		< .001
	≤ 24	228 (11.8)	64 (4.6)	292 (8.8)		212 (9.4)	62 (3.6)	274 (6.9)		567 (7.8)	
	25–35	1420 (73.6)	898 (64.6)	2318 (69.8)		1667 (73.7)	1056 (61.8)	2723 (68.6)		5043 (69.2)	
	≥ 36	281 (14.6)	428 (30.8)	709 (21.4)		382 (16.9)	591 (34.6)	973 (24.5)		1682 (23.1)	
	Missing	107 (5.*2)*	76 (5.2)	183 (5.2)		105 (4.4)	35 (2.0)	170 (4.1)		353 (4.6)	
Maternal Country of birth	Country born	1790 (94.*1)*	1253 (94.5)	3043 (94.2)	604	2031 (94.8)	1485 (93.7)	3516 (94.3)	.141	6561 (94.3)	.855
	Foreign	113 (5.*9)*	73 (5.5)	186 (5.8)		111 (5.2)	100 (6.3)	211 (5.7)		397 (5.7)	
	Missing	133 (6.5)	140 (9.6)	273 (7.8)		224 (9.5)	159 (9.0)	413 (10.0)		687 (9.0)	
Live with partner	Yes	1855 (93.0)	1351 (94.5)	3206 (93.7)	.073	2153 (93.1)	1635 (93.9)	3788 (93.4)	.291	6997 (93.5)	.699
	No	139 (7.0)	78 (5.5)	217 (6.3)		160 (6.9)	106 (6.1)	266 (6.6)		483 (6.5)	
	Missing	42 (2.*1)*	37 (2.5)	79 (2.3)		53 (2.2)	33 (1.9)	86 (2.1)		165 (2.2)	
Exposure to COVID-19	Yes	371 (18.2)	327 (22.3)	698 (19.9)	.003	463 (19.6)	446 (25.1)	909 (22.0)	< .001	1607 (21.0)	.031
	No	1665 (81.8)	1139 (77.7)	2804 (80.1)		1903 (80.4)	1328 (74.9)	3231 (78.0)		6038 (79.0)	
	Missing	0 (0.*0)*	0 (0.0)	0 (0.0)		0 (0.0)	0 (0.0)	0 (0.0)		0 (0.0)	
History of Mental Problems	Yes	532 (26.1)	382 (26.1)	914 (26.1)	.962	627 (26.5)	478 (26.9)	1105 (26.7)	.749	2019 (26.4)	.563
	No	1504 (73.9)	1084 (73.9)	2588 (73.9)		1739 (73.5)	1296 (73.1)	3035 (73.3)		5626 (73.6)	
	Missing	0 (0.0)	0 (0.0)	0 (0.0)		0 (0.0)	0 (0.0)	0 (0.0)		0 (0.0)	
					.001				.019		.224
	Secondary education or lower	480 (24.0)	417 (29.2)	897 (26.2)		549 (23.6)	465 (26.8)	1014 (25.0)		1911 (25.5)	
	Higher education	1521 (76.0)	1009 (70.8)	2530 (73.8)		1779 (76.4)	1270 (73.2)	3049 (75.0)		5581 (74.5)	
	Missing	35 (1.*7)*	40 (2.7)	75 (2.1)		38 (1.6)	39 (2.2)	77 (1.9)		153 (2.0)	
Unemployment					.280				.520		< .001
	Yes	362 (17.8)	240 (16.4)	602 (17.2)		322 (13.6)	229 (12.9)	551 (13.3)		1154 (15.1)	
	No	1674 (82.2)	1225 (83.6)	2899 (82.8)		2044 (86.4)	1543 (87.1)	3587 (86.7)		6488 (84.9)	
	Missing	0 (0.*0)*	1 (0.07)	1 (0.03)		0 (0.0)	2 (0.001)	2 (0.0005)		3 (0.0004)	
					< .001				< .001		< .001
	Albania	14 (0.7)	6 (0.4)	20 (0.6)		14 (0.6)	3 (0.2)	17 (0.4)		37 (0.5)	
	Brazil	124 (6.1)	144 (9.8)	268 (7.7)		328 (13.9)	269 (15.2)	597 (14.4)		865 (11.3)	
	Bulgaria	31 (1.5)	13 (0.9)	44 (1.3)		26 (1.1)	14 (0.8)	40 (1.0)		84 (1.1)	
	Chile	102 (5.0)	70 (4.8)	172 (4.9)		140 (5.9)	132 (7.4)	272 (6.6)		444 (5.8)	
	Cyprus	138 (6.8)	82 (5.6)	220 (6.3)		152 (6.4)	97 (5.5)	249 (6.0)		469 (6.1)	
	Greece	236 (11.6)	101 (6.9)	337 (9.6)		260 (11.0)	116 (6.5)	376 (9.1)		713 (9.3)	
	Israel	74 (3.6)	142 (9.7)	216 (6.2)		110 (4.6)	222 (12.5)	332 (8.0)		549 (7.2)	
	Malta	73 (3.6)	41 (2.8)	114 (3.3)		96 (4.1)	53 (3.0)	149 (3.6)		263 (3.4)	
	Portugal	441 (21.7)	312 (21.3)	753 (21.5)		385 (16.3)	284 (16.0)	669 (16.2)		1422 (18.6)	
	Spain	182 (8.9)	171 (11.7)	353 (10.1)		258 (10.9)	217 (12.2)	475 (11.5)		828 (10.8)	
	Turkey	524 (25.7)	282 (19.2)	806 (23.0)		407 (17.2)	213 (12.0)	620 (15.0)		1426 ( (18.7)	
	UK	97 (4.8)	102 (7.0)	199 (5.7)		190 (8.0)	154 (8.7)	344 (8.3)		545 (7.1)	

Missing values not included in calculation of percentages; *Pregnant *vs* postpartum women; ^1^one missing value on number of pregnancies for pregnant women; ^2^two missing values on number of pregnancies for postpartum women; UK, The United Kingdom.

Data from country-related variables are displayed in Table 2. Brazil and Turkey had the lowest IHDI, whereas Bulgaria and Malta had the lowest median CHI. Within the study period, the total deaths per million were highest for Spain, Brazil, and Chile.

**Table 2:** Country-related variables.

Country	Period of data collection	CHI				IHDI	Total deaths per million
		Median	P25	P75	Min–Max		Min–Max
Albania	15/07–23/08	56.70	56.50	56.77	55.52–56.82	0.708	35.10–86.87
Brazil	01/07–31/10	67.35	67.35	68.04	61.66–72.83	0.570	285.25–752.19
Bulgaria	12/06–30/10	39.63	39.23	39.76	37.21–46.69	0.721	24.75–180.47
Chile	02/10–31/10	70.95	70.95	70.95	70.95–70.95	0.709	673.09–743.19
Cyprus	23/06–29/10	53.99	53.80	54.72	50.83–62.87	0.805	21.69–28.54
Greece	23/06–31/10	51.37	49.25	54.31	47.52–59.31	0.791	18.23–60.06
Israel	01/07–29/10	64.87	52.68	65.54	39.66–72.92	0.814	38.13–291.72
Malta	03/07–31/10	46.53	45.80	49.02	39.91–53.02	0.823	20.38–140.42
Portugal	29/06–29/10	62.41	62.20	62.79	61.32–66.81	0.761	153.78–238.12
Spain	16/06–31/10	57.24	52.97	57.53	47.70–58.99	0.783	580.39–767.37
Turkey	22/06–31/10	60.65	53.26	66.97	49.39–67.57	0.683	58.98–121.56
UK	09/06–30/10	60.03	59.39	60.19	55.43–62.10	0.856	573.70–682.31

CHI, Containment and Health Index mean of the previous 30 days; IHDI, Inequality-adjusted Human Development Index; UK, The United Kingdom; P25, Percentile 25; P75, Percentile 75.

The prevalence of the overall clinically significant symptoms of depression and anxiety varied widely across countries (Table 3). The overall rate for clinically significant symptoms of depression in pregnant women was 26.8% (ranging from 18.2% in Cyprus to 37.7% in Brazil), and 32.7% (ranging from 17.6% in Albania to 47.2% in Brazil) in postpartum women. The prevalence of clinically significant anxiety symptoms in pregnant women was 20.1% (12.7% in Turkey to 36% in Chile) and 26.6% (ranging from 13.2% in Israel to 41.9% in Brazil) in postpartum women.

**Table 3:** Symptoms of depression and anxiety in pregnant and postpartum women by country.

Country	EPDS											
	Pregnant N = 3492		Postpartum N = 4131		Total N = 7623		Pregnant N = 3496		Postpartum N = 4121		Total N = 7617	
	Mdn (P_25_-P_75_)	EPDS ≥ 13 n (%)	Mdn (P_25_-P_75_)	EPDS ≥ 13 n (%)	Mdn (P_25_-P_75_)	EPDS ≥ 13 n (%)	Mdn (P_25_-P_75_)	GAD-7 ≥ 10 n (%)	Mdn (P_25_-P_75_)	GAD-7 ≥ 10 n (%)	Mdn (P_25_-P_75_)	GAD-7 ≥ 10 n (%)
Albania	8.0 (6.0–13.5)	5 (25.0)	8.0 (6.0–10.5)	3 (17.6)	8.0 (6.0–11.5)	8 (21.6)	5.0 (1.5–10.8)	7 (35.0)	5.0 (3.0–7.0)	3 (17.6)	5.0 (3.0–10.0)	10 (27.0)
Brazil	11.0 (7.0–16.0)	101 (37.7)	12.0 (8.0–17.0)	282 (47.2)	12.0 (8.0–16.0)	383 (44.3)	7.0 (4.0–12.8)	92 (34.3)	7.0 (5.0–13.0)	250 (41.9)	7.0 (4.5–13.0)	342 (39.5)
Bulgaria	8.0 (3.0–12.8)	11 (25.0)	7.0 (3.3–11.0)	9 (22.5)	7.5 (3.0–11.8)	20 (23.8)	5.0 (1.3–8.8)	9 (20.5)	4.5 (2.0–7.0)	6 (15.0)	5.0 (2.0–7.8)	15 (17.9)
Chile	9.0 (5.0–14.0)	60 (34.9)	11.0 (5.3–14.0)	92 (33.8)	10.0 (5.0–14.0)	152 (34.2)	7.0 (4.0–12.0)	62 (36.0)	8.0 (4.0–13.0)	113 (41.5)	7.5 (4.0–12.0)	175 (39.4)
Cyprus	6.0 (2.0–11.0)	40 (18.2)	7.0 (3.0–12.0)	56 (22.5)	7.0 (3.0–11.0)	96 (20.5)	3.5 (1.0–7.0)	30 (13.6)	4.0 (1.0–7.0)	40 (16.1)	4.0 (1.0–7.0)	70 (14.9)
Greece	6.0 (3.0–11.0)	64 (19.0)	7.0 (3.0–12.0)	90 (23.9)	7.0 (3.0–11.5)	154 (21.6)	6.0 (4.0–8.0)	51 (15.1)	6.0 (4.0–10.0)	96 (25.5)	6.0 (4.0–9.0)	147 (20.6)
Israel	7.0 (4.0–13.0)	59 (28.6)	7.0 (3.0–12.0)	67 (20.9)	7.0 (3.0–12.0)	126 (23.9)	4.0 (1.8–7.0)	33 (15.7)	3.0 (1.0–6.0)	41 (13.2)	4.0 (1.0–7.0)	74 (14.2)
Malta	9.0 (4.0–13.0)	31 (27.2)	9.0 (5.0–13.5)	51 (34.2)	9.0 (5.0–13.0)	82 (31.2)	5.0 (2.0–7.3)	21 (18.4)	5.0 (2.0–9.5)	37 (24.9)	5.0 (2.0–9.0)	58 (22.1)
Portugal	7.0 (4.0–12.0)	183 (24.3)	9.0 (5.0–13.0)	188 (28.1)	8.0 (4.0–13.0)	371 (26.1)	5.0 (3.0–8.0)	149 (19.8)	6.0 (3.0–9.0)	151 (22.6)	6.0 (3.0–9.0)	300 (21.1)
Spain	8.0 (4.0–13.0)	93 (26.3)	10.0 (5.0–14.0)	159 (33.5)	9.0 (5.0–13.0)	252 (30.4)	6.0 (3.0–11.0)	104 (29.5)	7.0 (3.0–12.0)	160 (33.7)	7.0 (3.0–11.0)	264 (31.9)
Turkey	8.0 (4.0–13.0)	225 (27.9)	9.0 (5.0–14.0)	216 (34.8)	9.0 (4.0–14.0)	441 (30.9)	3.0 (0.0–6.0)	102 (12.7)	4.0 (1.0–7.0)	101 (16.3)	3.0 (1.0–7.0)	203 (14.2)
UK^3^	9.0 (4.0–14.0)	63 (31.7)	11.0 (6.8–15.0)	136 (39.3)	10.0 (6.0–15.0)	199 (36.5)	4.0 (2.0–8.0)	42 (21.1)	6.0 (2.0–10.3)	97 (28.0)	5.0 (2.0–10.0)	139 (25.5)
Total	8.0 (4.0–13.0)	935 (26.8)	9.0 (5.0–14.0)	1349 (32.7)	9.0 (5.0–13.0)	2284 (30.0)	5.0 (2.0–8.0)	702 (20.1)	6.0 (3.0–10.0)	1095 (26.6)	5.0 (2.0–9.0)	1797 (23.6)

EPDS, Edinburgh Postnatal Depression Scale; GAD-7, the Generalised Anxiety Disorder Assessment; UK, The United Kingdom; Mdn, Median; P25, Percentile 25; P75, Percentile 75.

### Factors associated with symptoms of anxiety

**Figure 1:**
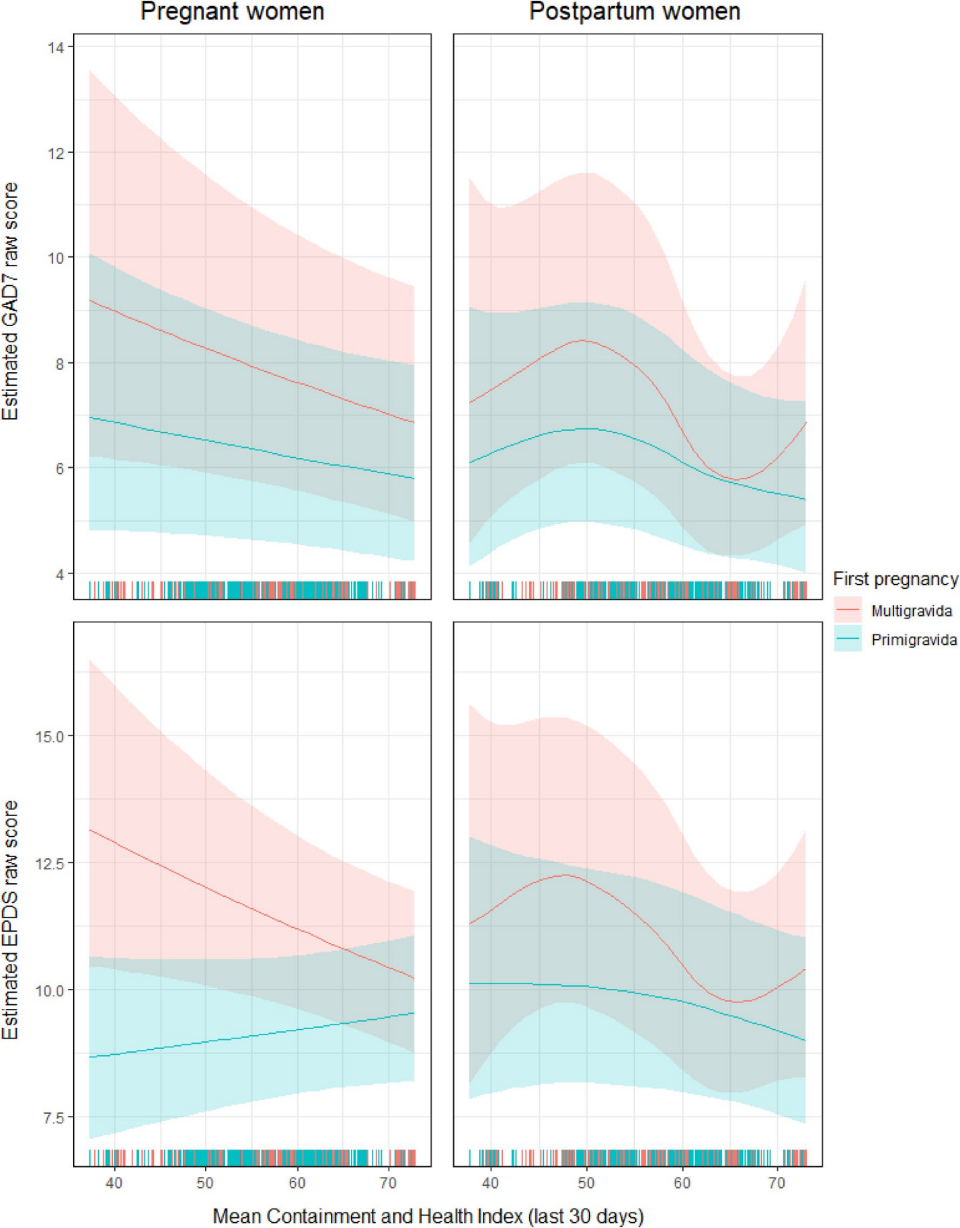
Estimated effect of Containment and Health Index scores on anxiety (top) and depression (bottom). The raw scores in pregnant (left) and postpartum women (right) are shown. Blue: primigravida, pink: multigravida.

**Figure 2:**
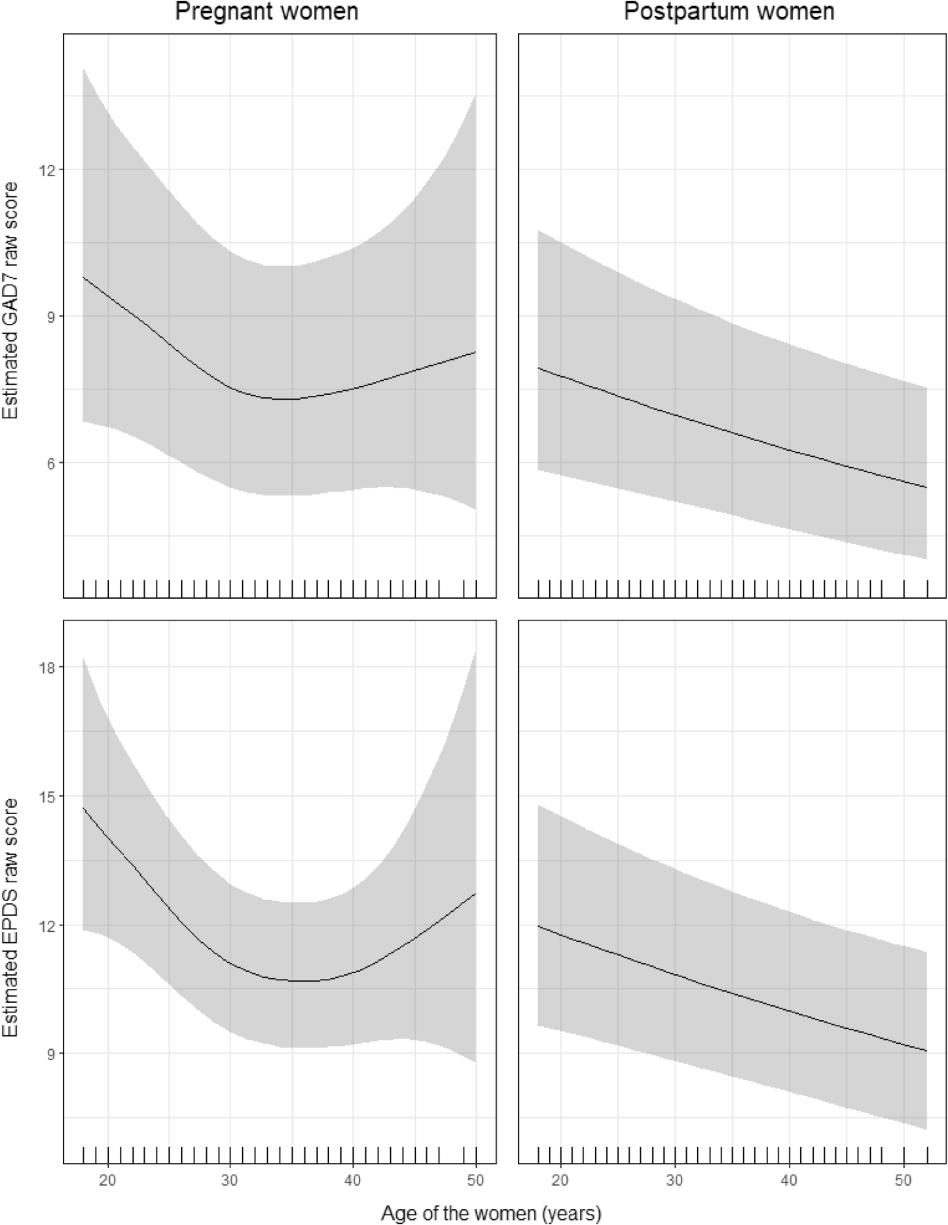
Estimated effect of the age of women on anxiety (top) and depression (bottom). The raw scores in pregnant (left) and postpartum women (right) are shown.

The results of the fixed effects of the generalised additive mixed models with 95% confidence intervals (CIs) are shown in Table 4. Exposure to COVID-19 and previous history of mental problems were associated with increased anxiety symptoms in pregnant and postpartum women. The total number of deaths per million due to COVID-19 was associated with increased anxiety symptoms in the postpartum women and living with the partner was associated with decreased anxiety in pregnant women. IHDI and CHI had a differential association with anxiety among pregnant and postpartum women. Multigravida tended to have higher anxiety in lower IHDI contexts. The association of anxiety with CHI was non-linear for postpartum women (Fig. 1). Symptoms of anxiety were highest for CHI around 50 (a score below the median for most of the countries in the data collection period) and lowest for CHI around 65 (a score close to the maximum score in most countries in the data collection period). Women’s age was associated with anxiety symptoms; this was a linear association for postpartum women (symptoms of anxiety decreased with age) and a non-linear association for pregnant women (lower anxiety symptoms in women aged between 30 and 40 years; Fig. 2). A sensitivity analysis excluding data from Albania, Bulgaria, and Malta yielded similar results (Supplementary Table 1; Supplementary Figs. 1 and 2).

**Table 4:** Multivariate regression models for GAD-7 and EPDS in pregnant and postpartum women. *p*-values lower than .05 are indicated in bold.

	Estimate (β)	Std. Error	Ratio (95%IC)	Edf	*P*-value
GAD7 Pregnant					
Intercept	3.345	0.648	28.353 (7.958, 101.040)	1.000	** < 0.001**
First Pregnancy					
Multigravida	0.000		1.000		
Primigravida	− 0.930	0.555	0.394 (0.133, 1.172)	1.000	0.094
COVID-19 Exposure					
No	0.000		1.000		
Yes	0.106	0.040	1.112 (1.029, 1.202)	1.000	**0.008**
Living with a partner					
No	0.000		1.000		
Yes	− 0.190	0.064	0.827 (0.730, 0.937)	1.000	**0.003**
History of Mental Problems					
No	0.000		1.000		
Yes	0.381	0.036	1.463 (1.364, 1.569)	1.000	** < 0.001**
Total deaths per million due to COVID-19 (hundreds)	0.027	0.017	1.027 (0.994, 1.061)	1.000	0.108
IHDI*First Pregnancy: Multigravida	− 1.452	0.694	0.234 (0.060, 0.913)	1.000	**0.036**
IHDI*First Pregnancy: Primigravida	− 0.502	0.687	0.605 (0.157, 0.238)	1.000	0.465
CHI_mean*First Pregnancy: Multigravida	− 0.008	0.004	0.992 (0.983, 1.001)	1.007	0.062
CHI_mean*First Pregnancy: Primigravida	− 0.005	0.004	0.995 (0.987, 1.003)	1.004	0.193
Age of the Mother			See Fig. 2	2.614	**0.002**
GAD7 Mothers					
Intercept	3.036	0.516	20.829 (7.582, 57.223)	1.000	** < 0.001**
First Pregnancy					0.114
Multigravida	0.000		1.000		
Primigravida	− 0.474	0.300	0.623 (0.346, 1.121)	1.000	0.114
COVID-19 Exposure					
No	0.000		1.000		
Yes	0.167	0.033	1.182 (1.109, 1.260)	1.000	** < 0.001**
Living with a partner					
No	0.000		1.000		
Yes	− 0.098	0.054	0.907 (0.816, 1.007)	1.000	0.067
History of Mental Problems					
No	0.000		1.000		
Yes	0.356	0.030	1.428 (1.347, 1.514)	1.000	** < 0.001**
Total deaths per million due to COVID-19 (hundreds)	0.051	0.015	1.052 (1.022, 1.082)	1.000	** < 0.001**
IHDI*First Pregnancy: Multigravida	− 1.368	0.659	0.255 (0.070, 0.926)	1.000	**0.038**
IHDI*First Pregnancy: Primigravida	− 0.736	0.638	0.479 (0.137, 1.671)	1.000	0.248
CHI_mean*First Pregnancy: Multigravida			See Fig. 1	4.098	** < 0.001**
CHI_mean*First Pregnancy: Primigravida				2.488	0.058
Age of the Mother	− 0.011	0.003	0.989 (0.984, 0.995)	1.005	** < 0.001**
EPDS Pregnant					
Intercept	3.582	0.350	35.946 (18.085, 71.447)	1.000	** < 0.001**
First Pregnancy					
Multigravida	0.000		1.000		
Primigravida	− 1.266	0.450	0.282 (0.117, 0.681)	1.000	**0.005**
COVID-19 Exposure					
No	0.000		1.000		
Yes	0.101	0.032	1.106 (1.038, 1.178)	1.000	**0.002**
Living with a partner					
No	0.000		1.000		
Yes	− 0.163	0.052	0.849 (0.767, 0.940)	1.000	**0.002**
History of Mental Problems					
No	0.000		1.000		
Yes	0.331	0.029	1.392 (1.316, 1.473)	1.000	** < 0.001**
Total deaths per million due to COVID-19 (hundreds)	0.012	0.006	1.012 (0.999, 1.024)	1.000	0.051
IHDI*First Pregnancy: Multigravida	− 1.304	0.296	0.272 (0.152, 0.485)	1.000	** < 0.001**
IHDI*First Pregnancy: Primigravida	− 0.449	0.287	0.638 (0.364, 1.121)	1.000	0.118
CHI_mean*First Pregnancy: Multigravida	− 0.007	0.003	0.993 (0.987, 0.999)	1.006	**0.025**
CHI_mean*First Pregnancy: Primigravida	0.003	0.003	1.003 (0.997, 1.008)	1.008	0.327
Age of the Mother			See Fig. 2	2.844	** < 0.001**
EPDS Mothers					
Intercept	3.298	0.351	27.051 (13.593, 53.834)	1.000	** < 0.001**
First Pregnancy					
Multigravida	0.000		1.000		
Primigravida	0.439	0.227	0.645 (0.413, 1.007)	1.000	0.054
COVID-19 Exposure					
No	0.000		1.000		
Yes	0.082	0.026	1.086 (1.032, 1.142)	1.000	**0.002**
Living with a partner					
No	0.000		1.000		
Yes	− 0.099	0.043	0.905 (0.833, 0.985)	1.000	**0.020**
History of Mental Problems					
No	0.000		1.000		
Yes	0.278	0.024	1.321 (1.009, 1.384)	1.000	** < 0.001**
Total deaths per million due to COVID-19 (hundreds)	0.029	0.010	1.029 (1.009, 1.050)	1.000	**0.005**
IHDI*First Pregnancy: Multigravida	− 1.184	0.446	0.306 (0.128, 0.734)	1.000	**0.008**
IHDI*First Pregnancy: Primigravida	− 0.581	0.422	0.559 (0.245, 1.279)	1.000	0.169
CHI_mean*First Pregnancy: Multigravida			See Fig. 1	3.369	**0.003**
CHI_mean*First Pregnancy: Primigravida				1.486	0.267
Age of the Mother	− 0.008	0.002	0.992 (0.987, 0.996)	1.019	** < 0.001**

GAD-7, seven-item Generalised Anxiety Disorder Assessment; IHDI, Inequality-adjusted human developmental index; CHI, Containment and health index; EPDS, Edinburgh Postnatal Depression Scale; COVID-19, coronavirus disease 2019; SE: standard error.

### Factors associated with symptoms of depression

The results of the fixed effects of the generalised additive mixed models are shown in Table 4. Symptoms of depression were lower for primigravida compared to multigravida for pregnant women. COVID-19 exposure, not living with a partner and previous history of mental problems and the total number of national deaths per million were associated with increased depression in pregnant and postpartum women. Total number of national deaths per million were associated with increased depression in postpartum women.

Both IHDI and CHI were associated with symptoms of depression in multigravida women. Specifically, symptoms of depression increased in lower IHDI contexts in both pregnant and postpartum women and in lower CHI contexts only in pregnant women. The association between symptoms of depression and CHI in postpartum women was non-linear (Fig. 1). Symptoms of depression were highest for CHI around 47 (a score below the median for most of the countries in the data collection period) and lowest for CHI around 66 (a score close to the maximum score in most countries in the data collection period). Women’s age was associated with depression symptoms; this was a linear association for postpartum women (symptoms of depression decreased with age) and a non-linear association for pregnant women (lower symptoms of depression in women aged between 30 and 40 years; Fig. 2). A sensitivity analysis excluding data from Albania, Bulgaria, and Malta yielded similar results (Supplementary Table 1; Supplementary Figs. 1 and 2).

## Discussion

Overall, our data demonstrated that during the COVID-19 pandemic, one in four women during pregnancy and one in three women in the postpartum had clinically significant symptoms of depression. One in five women during pregnancy and one in four women in the postpartum had clinically significant symptoms of anxiety. Additionally, more robust governmental responses to the COVID-19 outbreak, measured by the CHI, were associated with lower symptoms of anxiety in the postpartum period and depression during the perinatal period. Furthermore, previous history of mental health problems and exposure to COVID-19 were important determinants of anxiety and depression regardless of the perinatal period. Individual determinants such as gravidity were associated specifically with depression in pregnancy and living with the partner showed to be protective of mental health problems during pregnancy and protective of depression during the postpartum period. National deaths per million due to COVI D-19 was associated with mental health problems only in the postpartum. Furthermore, multigravida women from countries with lower IHDI or CHI were more likely to have higher symptoms of depression or anxiety in the postpartum period. Moreover, symptoms of depression and anxiety decreased with age in postpartum women, whereas during pregnancy, lower symptoms of depression and anxiety were observed between the ages of 30 and 40 years.

### Prevalence of clinically significant symptoms of anxiety and depression

The overall rates of clinically significant symptoms of depression are alarming and, although there are variations between countries, even in countries with lower rates—Cyprus, Greece, and Albania—at least one in four women had clinically significant symptoms of depression. In 6/12 countries, the rates were above 30%, including Spain, Turkey, Malta, Chile, the United Kingdom (UK), and Brazil. Other studies conducted during the COVID-19 pandemic reported even higher rates of clinically significant depressive symptoms during pregnancy (37%) using the same instrument and cut-off^32^. Nonetheless, our rates are higher than those found in a cross-country study conducted during the COVID-19 pandemic (June to July 2020) in Ireland, Norway, Switzerland, the Netherlands, and the UK, in which rates of clinically significant depression symptoms were about 15% for pregnant women and 10% for postpartum women^33^. Although in our study Spain is among the countries with higher rates of clinically significant depression symptoms (30.4%), even higher rates were reported in a recent study (58%) conducted by Chavez et al.^34^ in which an EPDS cut-off of 11 was used instead of 13, increasing the number of individuals scoring above the threshold. Turkey also had high rates of clinically significant depression symptoms, mirroring the results of a recent study showing a prevalence of 35.4% among pregnant women in Turkey^35^. In the UK, Fallon et al.^15^ reported slightly higher rates in postpartum women (43.0%) than in our study (39.3%). In Brazil, a recent study with a cohort of women in the postpartum period from a city located in the south of the country reported that 29.3% clinically significant symptoms of depression^36^. While this rate is lower than reported in our study (47.2%), it should be noted that it pertains to a specific city and that some women were assessed over the 12-month postpartum period when peak symptoms occur.

In three-quarters of the countries (9/12) evaluated in this study, at least one in five women displayed clinically significant anxiety symptoms, lower than the frequency of clinically significant depression symptoms in all countries except Albania, Chile, and Spain. Other researchers have found higher rates of clinically significant symptoms of anxiety compared to depression during pregnancy in the pandemic period, for instance, in Canada (57–37%), Iran (43.9–32.7%) or China (31.2–19.2%)^23–38^, which concurs with our data regarding some countries (Albania, Chile, and Spain). Whether cultural or COVID-19-related factors are responsible for these differences remains to be understood since we lack pre-pandemic data. In the current study, the rates of clinically significant anxiety and depression symptoms were higher in the postpartum period than during pregnancy in most countries except Albania, Bulgaria, and Israel (and Chile, regarding depression only), which indicates that postpartum women are at a higher risk of mental health problems then pregnant women, as previously reported^39^. This evidence highlights the importance of implementing responsive and adequate mental health services for postpartum women to overcome barriers to care during this period (e.g., availability of childcare facilities/staff and breastfeeding support).

### Individual Factors associated with depression and anxiety

Individual determinants such as previous history of mental health problems are important determinants of anxiety and depression regardless of the perinatal period. Pre-existing mental health conditions are a well -known risk factor for perinatal mental health problems and have been associated with higher levels of anxiety and depressive symptoms in other studies conducted during the COVID-19 pandemic in the general population^40^ and in the perinatal period^41^.

Aditionally, living with the partner seems to be protective of both anxiety and depression during pregnancy and protective of depression during the postpartum period, in line with previous evidence^42^.This may be due to the fact that social support is protective of perinatal mental health^18^ and partner support is perceived by pregnant women as one of the most important sources of social support during the COVID-19 pandemic^14^. Moreover, gravidity is associated specifically with depression in pregnancy, which is in line with other studies and may be explained by the fact that multiparous women are exposed to additional burden related to their pre-existing parenting challenges^11^. Additionally, symptoms of depression and anxiety decreased with age in postpartum women, whereas during pregnancy, lower symptoms of depression and anxiety were observed between the ages of 30 and 40 years. Others studies have consistently associated younger ages with mental health problems among women in the perinatal period^18,33^. The higher scores for depression and anxiety in older women during pregnancy but not in the postpartum period, might be related with the cumulative threat of the COVID-19 disease and of the age by itself that is a well-known risk factor for perinatal and neonatal outcomes.

### Impact of governmental responses to COVID-19 outbreak

Governmental responses to control the spread of COVID-19 differed between countries. Increased CHI was associated with decreased symptoms of depression or anxiety during the postpartum period; thus, the number and intensity of closure and containment policies and those aimed at disease surveillance were protective of women’s perinatal mental health. Other studies conducted during the first phase of the COVID-19 pandemic, also showed decreased depression and anxiety symptoms with more strict restriction measures^40^, as well as a positive perception on lockdowns and improvements in perceived mental well-being^43^ in the general population. Still, conflicting evidence highlight the potentially negative impact of such restrictive measures (namely social isolation) and the potential burden in terms of mental health outcomes^44–47^.

These apparently contradictory results should be carefully interpreted since there are several methodological differences between studies. In our study we used a cross-country comparable Government Response Tracker (OxCGRT), an index based on several specified indicators capturing variation in the restriction level of governmental responses, while other’s studies^14,15^ have relied on self-report questionnaires to assess individual perceptions of restrictions particularly focused on isolation and social support/distance. One explanation for our results could be that the COVID-19 pandemic represents uncertainty (at the social, economic, and health levels) and that these governmental interventions may be perceived as protective, especially for vulnerable populations such as perinatal women, reassuring individuals and providing them with a sense of security and control over this challenging situation^22^.

Interestingly, multigravida women were the most ‘sensitive’ to both the governmental measures and to the IHDI. Regarding CHI, the pattern found for multigravida women is different for the one found in primigravida women which could be explained by the additional parenting challenges for multigravida. Some studies showed that the COVID-19 pandemic was associated with parenting-related exhaustion particularly for women or having higher number of children or younger children^48^.

We also observed that women living in countries with high IHDI displayed lower symptoms of anxiety and depression. Every increase of 0.1 points on the IHDI led to a decrease of 10.4–13.9% in anxiety in pregnant women and in depression in both pregnant and postpartum women. This finding contrasts with the higher depression symptoms in countries with higher HDI reported in a systematic review (Lee et al., 2021). However, in those studies, the HDI was used, whereas in our study the IHDI was used. Our data highlight that exposure to multiple risk factors (characteristic of countries with higher inequalities) is associated with increased vulnerability to poor mental health. In the context of COVID-19, women in the perinatal period from resource-constrained countries may be at even higher risk of poor mental health than those countries with less inequality.

Symptoms of anxiety and depression increased with the magnitude of the pandemic’s negative effects, such as the number of deaths and exposure to COVID-19. For every 100 deaths per million, the depressive symptoms increased between 2.7 and 3.7%, and the anxiety symptoms increased between 4.4 and 5.9%. These data support the assumption that COVID-19 may lead to national mental health crises, particularly in countries with greater infection levels^49^. Additionally, COVID-19 exposure led to an 8.7% increase in anxiety symptoms and an 18.6% increase in symptoms of depression. Previous evidence showed that having symptoms suggestive of COVID-19 was a risk factor for mental health problems in women^50^.

The highest prevalence of clinically significant depression symptoms was observed in Brazil (44.3%), Chile (34.2%), and the UK (35.5%), which were the three countries with the greatest delays in the implementation of stringent lockdown measures. Considering a threshold of 20 in the governments’ stringent COVID-19 containment index^51^, Brazil, Chile, and the UK took 6, 9, and 12 more days, respectively, than the leading countries (Israel, Greece, and Bulgaria), to implement the same level of stringency. Previous studies indicated that symptoms of depression were higher in countries implementing these measures later^51^.

### Strengths and limitations

The main strength of this study is the use of reliable self-reported measures in 12 countries. The main strength of this study is the use of reliable self-reported outcome measures in 12 countries. Both the EPDS and GAD-7, are widely used in research and have good properties to estimate the prevalence of depression and anxiety^28,30^. However, there are noteworthy limitations to this study. First, even though the gold standard for the assessment of clinical prevalence are structured clinical interviews, applying structured interviews in large samples, and especially during the COVID-19 pandemic wouldn’t be feasible. Second, although the GAD-7^30^ is not validated for perinatal population, this screening tool appears to capture the symptomatology and severity of the illness in pregnant and postpartum mothers^52^ and it is recommended by the NICE 2020 clinical guidelines^53^. Third, not all countries included in the present study have validated the EPDS in the perinatal period, whereas validation studies for using GAD-7 in the perinatal period are lacking for most of these countries^54,55^. Therefore, further research is needed to determine the validity and reliability of these screening tools during the perinatal period across different countries.

Although we could not report the impact of national inequalities on mental health, future studies with data from countries representing a wider range of IHDI sub-groups could provide further details on the impact of inequalities on perinatal mental health.

The fact that most of the participants had high levels of education is a limitation of this study. It may lead to underestimating mental health problems since a higher educational level is a protective factor for mental health problems^56^ and may limit the generalizability of the results. Similarly, women under 18 years old, who may be especially vulnerable to the adverse effects of the COVID-19 pandemic, were not included in the study due to ethical issues. Additionally, we do not have socio-demographic information for women excluded due to missing data, therefore we cannot compare potential differences between women included and not included in the analysis.

It is also important to note that this study reports data collected between June and October 2020, before the second wave of COVID-19 in Europe, may explain the lower rates of clinically significant symptoms of depression and anxiety in our study, specifically in the European countries, compared with other studies conducted early on in the pandemic. Additionally, the long-term impact of prolonged exposure to such restrictive governmental measures on perinatal mental health remains to be investigated.

## Conclusion

In conclusion, this study showed that containment health measures are associated with better prenatal mental health outcomes in the initial period of the pandemic; while in the postnatal period a non-linear effect was observed. Country-related and individual-related factors explain some of the variability in this association, with women from more disadvantaged countries at higher risk for adverse mental health outcomes. Implementing more restrictive measures seems to be especially protective in multigravida women.

A global investment in perinatal mental health care policies is urgent in order to mitigate the long-term impact of COVID-19 on women, children and families. Specific measures for vulnerable groups such as multiparous women and those from low-resources countries, should be implemented by policy makers.

## Supplementary Information

Supplementary Information 1.

Supplementary Information 2.

Supplementary Information 3.

## Data Availability

The datasets analysed during the current study are available from the corresponding authors on reasonable request.
