# Supplementary material for: A cross-country study on the impact of governmental responses to the COVID-19 pandemic on perinatal mental health
Source: Sci Rep. 2023 Feb 16;13:2805. doi: 10.1038/s41598-023-29300-w (PMC9933810; doi:10.1038/s41598-023-29300-w)
Supplement: Supplementary file 1 — Supplementary Information 1. [file 41598_2023_29300_MOESM1_ESM.docx]

|  |  |  | **Estimate (β)** | **Std. Error** | **Ratio (95%IC)** | **Edf** | **P-value** |
| --- | --- | --- | --- | --- | --- | --- | --- |
|  |  | **GAD7 Pregnant** |  |  |  |  |  |
|  |  | Intercept | 3.278 | 0.732 | 26.538 (6.318, 111.472) | 1.000 | <0.001 |
|  |  | First Pregnancy |  |  |  |  |  |
|  |  | Multigravida | 0.000 |  | 1.000 |  |  |
|  |  | Primigravida | -0.839 | 0.595 | 0.432 (0.135, 1.387) | 1.000 | 0.159 |
|  |  | COVID-19 Exposure  No  Yes | 0.000  0.101 | 0.040 | 1.000  1.107 (1.022, 1.198) | 1.000 | 0.012 |
|  |  | Living with a partner  No  Yes | 0.000  -0.202 | 0.067 | 1.000  0.817 (0.716, 0.932) | 1.000 | 0.003 |
|  |  | Previous History of Mental Problems  No  Yes | 0.000  0.390 | 0.036 | 1.000  1.478 (1.376, 1.587) | 1.000 | <0.001 |
|  |  | Total deaths per million due to COVID-19 (hundreds) | 0.026 | 0.018 | 1.026 (0.990, 1.064 | 1.000 | 0.157 |
|  |  | IHDI*First Pregnancy: Multigravida | -1.398 | 0.788 | 0.247 (0.053, 1.159) | 1.000 | 0.076 |
|  |  | IHDI*First Pregnancy: Primigravida | -0.455 | 0.786 | 0.635 (0.136, 2.962) | 1.000 | 0.563 |
|  |  | CHI_mean*First Pregnancy: Multigravida | -0.008 | 0.005 | 0.992 (0.983, 1.002) | 1.012 | 0.104 |
|  |  | CHI_mean*First Pregnancy: Primigravida | -0.006 | 0.004 | 0.994 (0.985, 1.002) | 1.002 | 0.159 |
|  |  | Age of the Mother |  |  | See Figure 2 | 2.660 | 0.001 |
|  |  | **GAD7 Mothers** |  |  |  |  |  |
|  |  | Intercept | 3.017 | 0.571 | 20.423 (6.675, 62.484) | 1.000 | <0.001 |
|  |  | First Pregnancy  Multigravida  Primigravida | 0.000  -0.417 | 0.293 | 1.000  0.659 (0.371, 1.171) | 1.000 | 0.155 |
|  |  | COVID-19 Exposure  No  Yes | 0.000  0.175 | 0.033 | 1.000  1.191 (1.116, 1.270) | 1.000 | <0.001 |
|  |  | Living with a partner  No  Yes | 0.000  -0.078 | 0.055 | 1.000  0.925 (0.830, 1.031) | 1.000 | 0159 |
|  |  | Previous History of Mental Problems  No  Yes | 0.000  0.353 | 0.030 | 1.000  1.423 (1.341, 1.511) | 1.000 | <0.001 |
|  |  | Total deaths per million due to COVID-19 (hundreds) | 0.052 | 0.016 | 1.053 (1.021, 1.087) | 1.000 | 0.001 |
|  |  | IHDI*First Pregnancy: Multigravida | -1.391 | 0.730 | 0.249 (0.059, 1.041) | 1.000 | 0.057 |
|  |  | IHDI*First Pregnancy: Primigravida | -0.830 | 0.699 | 0.436 (0.111, 1.715) | 1.000 | 0.235 |
|  |  | CHI_mean*First Pregnancy: Multigravida |  |  | See Figure 1 | 4.082 | <0.001 |
|  |  | CHI_mean*First Pregnancy: Primigravida |  |  |  | 1.042 | 0.014 |
|  |  | Age of the Mother | -0.011 | 0.003 | 0.989 (0.984, 0.995) | 1.003 | <0.001 |
|  |  | **EPDS Pregnant** |  |  |  |  |  |
|  |  | Intercept | 3.485 | 0.368 | 32.613 (15.858, 67.067) | 1.000 | <0.001 |
|  |  | First Pregnancy  Multigravida  Primigravida | 0.000  -1.280 | 0.484 | 1.000  0.278 (0.108, 0.719) | 1.000 | 0.008 |
|  |  | COVID-19 Exposure  No  Yes | 0.000  0.095 | 0.033 | 1.000  1.100 (1.031, 1.173) | 1.000 | 0.004 |
|  |  | Living with a partner  No  Yes | 0.000  -0.156 | 0.055 | 1.000  0.856 (0.768, 0.954) | 1.000 | 0.005 |
|  |  | Previous History of Mental Problems  No  Yes | 0.000  0.330 | 0.029 | 1.000  1.391 (1.313, 1.474) | 1.000 | <0.001 |
|  |  | Total deaths per million due to COVID-19 (hundreds) | 0.013 | 0.006 | 1.013 (1.002, 1.024) | 1.000 | 0.023 |
|  |  | IHDI*First Pregnancy: Multigravida | -1.282 | 0.292 | 0.278 (0.157, 0.492) | 1.000 | <0.001 |
|  |  | IHDI*First Pregnancy: Primigravida | -0439 | 0.283 | 0.645 (0.370, 1.123) | 1.000 | 0.121 |
|  |  | CHI_mean*First Pregnancy: Multigravida | -0.006 | 0.003 | 0.994 (0.987, 1.001) | 1.003 | 0.087 |
|  |  | CHI_mean*First Pregnancy: Primigravida | 0.004 | 0.003 | 1.004 (0.998, 1.010) | 1.482 | 0.164 |
|  |  | Age of the Mother |  |  | See Figure 2 | 2.913 | <0.001 |
|  |  | **EPDS Mothers** |  |  |  |  |  |
|  |  | Intercept | 3.383 | 0.355 | 29.464 (14.695, 59.076) | 1.000 | <0.001 |
|  |  | First Pregnancy  Multigravida  Primigravida | 0.000  -0.343 | 0.231 | 1.000  0.710 (0.450, 1.118) | 1.000 | 0.139 |
|  |  | COVID-19 Exposure  No  Yes | 0.000  0.084 | 0.026 | 1.000  1.088 (1.033, 1.146) | 1.000 | 0.001 |
|  |  | Living with a partner  No  Yes | 0.000  -0.087 | 0.045 | 1.000  0.917 (0.840, 1.001) | 1.000 | 0.052 |
|  |  | Previous History of Mental Problems  No  Yes | 0.000  0.276 | 0.024 | 1.000  .317 (1.256, 1.382) | 1.000 | <0.001 |
|  |  | Total deaths per million due to COVID-19 (hundreds) | 0.028 | 0.010 | 1.028 (1.008, 0.049) | 1.000 | 0.006 |
|  |  | IHDI*First Pregnancy: Multigravida | -1.313 | 0.451 | 0.269 (0.111, 0.651) | 1.000 | 0.004 |
|  |  | IHDI*First Pregnancy: Primigravida | -0.842 | 0.431 | 0.431 (0.185, 1.003) | 1.000 | 0.051 |
|  |  | CHI_mean*First Pregnancy: Multigravida |  |  | See Figure 1 | 3.449 | 0.001 |
|  |  | CHI_mean*First Pregnancy: Primigravida |  |  |  | 1.670 | 0.227 |
|  |  | Age of the Mother | -0.008 | 0.002 | 0.992 (0.987, 0.997) | 1.033 | <0.001 |

Table 1: Multivariate regression models for GAD7 and EPDS in pregnant women and mothers. P-values lower that 0.05 are indicated in red.
