# Supplementary material for: A cross-country study on the impact of governmental responses to the COVID-19 pandemic on perinatal mental health
Source: Sci Rep. 2023 Feb 16;13:2805. doi: 10.1038/s41598-023-29300-w (PMC9933810; doi:10.1038/s41598-023-29300-w)
Supplement: Supplementary file 2 — Supplementary Information 2. [file 41598_2023_29300_MOESM2_ESM.pdf]

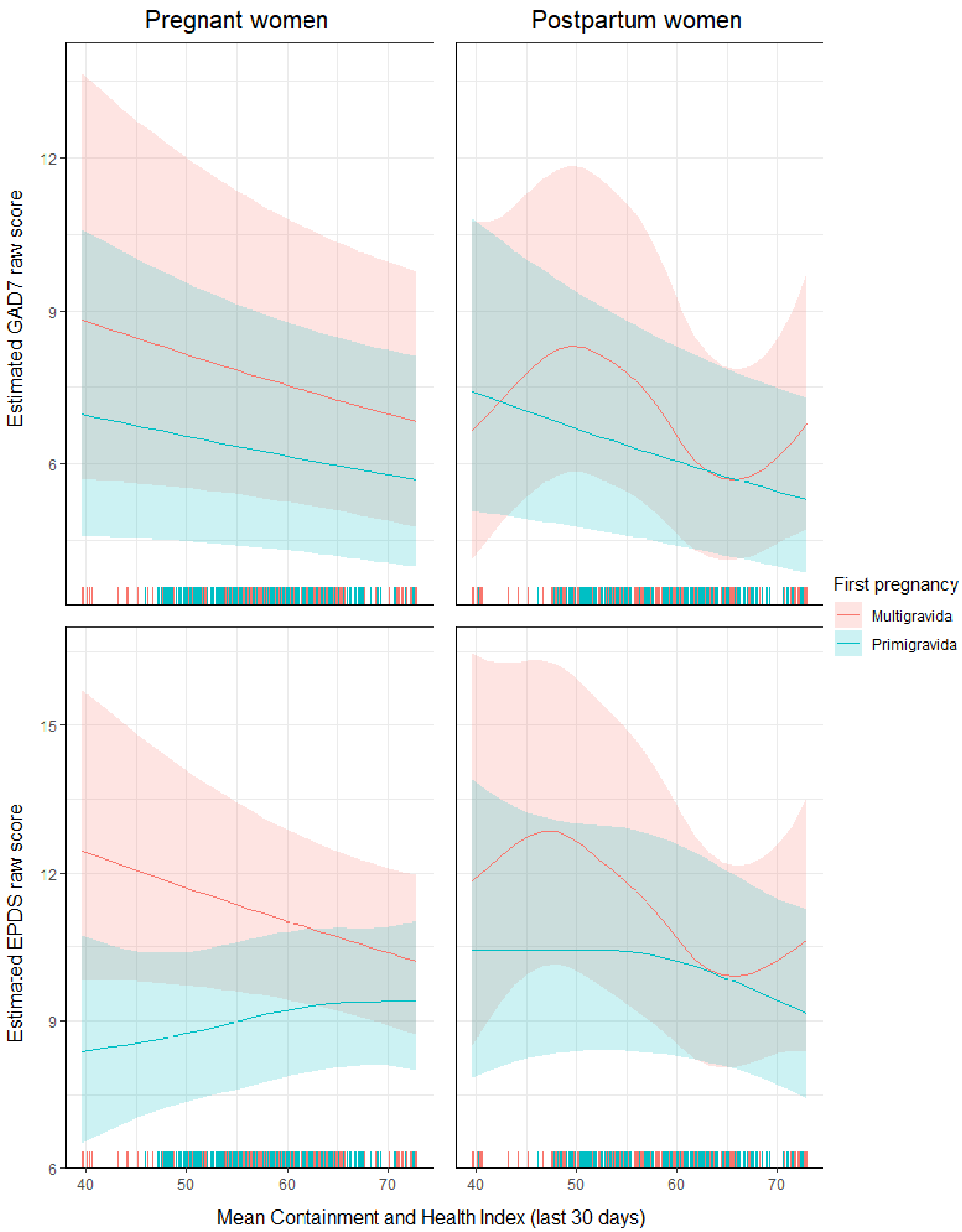

Supplementary Figur e 1. Sensitivity analysis excludi ng Al bania, Bulgaria, and Malta. Estimated effect of Containment and Health Index scores on anxiety (top) and depression (bottom) raw scores i n pregnant (left) and postpartum women (right) in primigravida (blue) and multigravida (pink).
