# Supplementary material for: A cross-country study on the impact of governmental responses to the COVID-19 pandemic on perinatal mental health
Source: Sci Rep. 2023 Feb 16;13:2805. doi: 10.1038/s41598-023-29300-w (PMC9933810; doi:10.1038/s41598-023-29300-w)
Supplement: Supplementary file 3 — Supplementary Information 3. [file 41598_2023_29300_MOESM3_ESM.pdf]

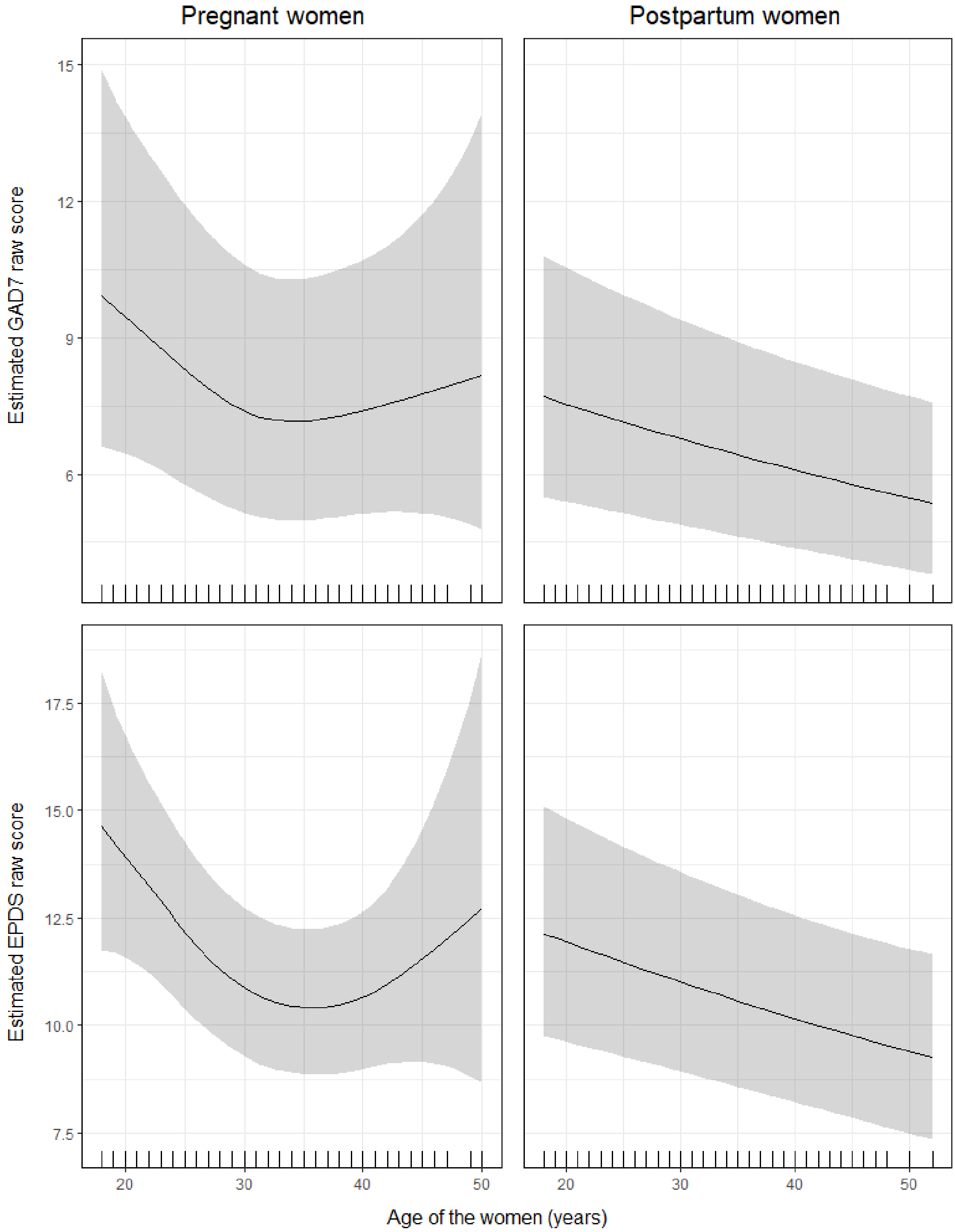

Supplementary Figure 2. Sensitivity analysis excluding Al bania, Bulgaria and Malta. Estimated effect of the age of women on anxiety (top) and depr ession (bottom) raw scores in pregnant (left) and postpartum women (right).
